# Spontaneous dissection of the renal artery: case report

**DOI:** 10.1590/1677-5449.009917

**Published:** 2018

**Authors:** Marcio Miyamotto, Carla Mariko Okabe, Paulo Roberto Pancheniak Neumann, Bruna Da Lozzo, Giana Caroline Strack Neves, Cintia Lopes Raymundo

**Affiliations:** 1 Pontifícia Universidade Católica do Paraná – PUC-PR, Hospital Universitário Cajuru – HUC, Serviço de Cirurgia Vascular e Endovascular, Curitiba, PR, Brasil.; 2 Instituto VESSEL de Aperfeiçoamento Endovascular de Curitiba, Curitiba, PR, Brasil.; 3 Hospital Nossa Senhora das Graças – HNSG, Serviço de Cirurgia Vascular e Endovascular Elias Abrão, Curitiba, PR, Brasil.; 4 Pontifícia Universidade Católica do Paraná – PUC-PR, Hospital Universitário Cajuru – HUC, Liga Acadêmica de Medicina Vascular – LAMEV, Curitiba, PR, Brasil.

**Keywords:** renal artery, dissection, angioplasty

## Abstract

Spontaneous renal artery dissection is rare and most cases are considered idiopathic. Previous renal arterial disease may be present in some cases and clinical presentation is often non-specific. Here, the authors present a case of spontaneous renal artery dissection in a 40-year-old male patient with uncontrolled hypertension discovered during investigation of secondary hypertension. Duplex ultrasound initially showed 80% left renal artery stenosis which was shown to be a renal artery dissection during angiography. The patient was successfully managed by percutaneous placement of a renal artery stent.

## INTRODUCTION

 Spontaneous dissection of the renal artery (SDRA) was first described by Bumpus in 1944 1 and is a rare pathology, defined as dissection with no relation to prior traumas or arterial interventions. 2 A significant proportion of cases involve people with no previously known vascular conditions, although there may also be associations with atherosclerosis, intimal fibroplasia, malignant hypertension, Marfan Syndrome, Ehlers-Danlos Syndrome, or intense physical effort. 3

 Here, the authors describe the case of a patient diagnosed with SDRA unrelated to prior renal disorders, who was treated with endovascular stent placement. 

## CASE REPORT

**Figure 1:**
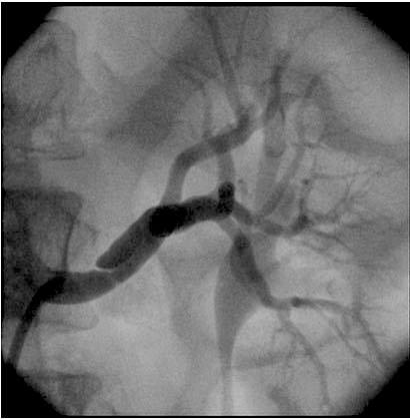
Arteriography showing stenosis in the mid distal third of the renal artery.

**Figure 2:**
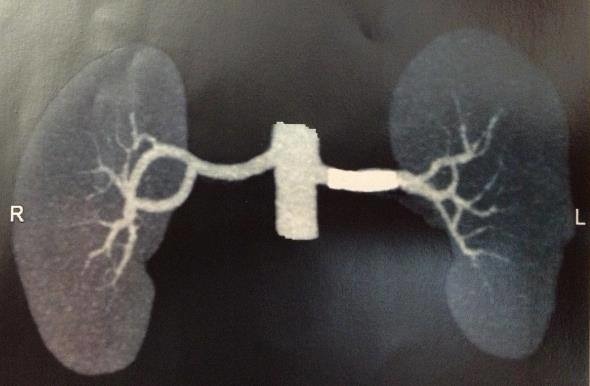
Control angiotomography after placement of stent.

 A male, 40-year-old patient presented with sudden exacerbation of arterial hypertension that had hitherto been controlled with two drugs (amlodipine and valsartan). Renal function was preserved, with no abnormalities, and urea and creatinine levels were also normal. He had a prior history of chronic hepatitis and smoking (20 pack years). Investigation of probable secondary hypertension was initiated with Doppler ultrasonography of the renal arteries, revealing stenosis (> 80%) of the mid third of the left renal artery. The Doppler ultrasonography images were considered satisfactory and compatible with the patient’s clinical status. In addition to providing images, the Doppler ultrasonography findings enabled the degree of stenosis to be calculated in terms of anatomic criteria and velocity, and provided sufficient evidence to indicate angiography and treatment planning during a single procedure. Angiography revealed dissection of the renal artery, with double lumen and reduction in the vessel’s caliber, provoking stenosis of the segment ( Figure 1 ). The patient was treated by placement of two stents, one a longer self-expanding stent and the other a balloon-expanded covered stent along the zone of dissection. Clinical progress was good and it proved possible to control arterial hypertension with just one drug. A control angiotomography at 3 months showed that the dissection had been resolved and the stents were patent ( Figure 2 ). 

## DISCUSSION

 Spontaneous renal artery dissection may occur spontaneously or secondary to local changes involving the renal artery, such as atherosclerosis, intimal fibroplasia, Marfan Syndrome, and Ehlers-Danlos Syndrome, or even malignant hypertension or intense physical activity. 3 ^,^ 4 In this case, arterial disorders compatible with fibromuscular dysplasia of the renal arteries and other vessels were investigated, as were other associated vascular diseases, but no such abnormalities were detected. While the etiopathogenesis of this disease remains uncertain, many authors suggest that there it involves rupture of the tunica intima or formation of an intramural hematoma due to rupture of the vasa vasorum. 5

 Spontaneous renal artery dissection predominantly occurs in males (the ratio of men to woman can range from 4:1 to 10:1), who are generally smokers in the fourth to sixth decades of their lives. 5 ^-^ 8 The patient in the case described here fits this profile. 

 In the acute phase, clinical presentation of SDRA can be nonspecific, manifesting as lumbar pain that can simulate renal colic or even pain with muscular origin, although the majority of patients are asymptomatic. Pain can also be secondary to renal infarction caused by abrupt occlusion of segmental branches or even the primary renal artery. In later stages, the patient may present renal ischemia manifesting as renovascular hypertension, as in the case described here. 9 When our patient was interviewed afterwards, to investigate factors that could be related to dissection of the renal artery, he described an episode of nonspecific lumbar pain on the right side. At the time he had attributed the pain to a distended muscle caused by trauma during martial arts practice (jiu-jitsu). He mentioned that the onset of pain had not been temporally related to the martial arts sessions. Another relevant factor is that he had not suffered a trauma of sufficient intensity to cause dissection of the renal artery secondary to blunt trauma. The heterogeneous and nonspecific presentation may cause confusion and lead to delays in recognition and treatment of this entity and more than half of these patients already have renal infarction at the time of diagnosis. 9

 Angiography is recognized as the definitive diagnostic method for renal artery dissections, because it precisely shows the extent and nature of arterial involvement and can also identify associated disorders. 10 Considering the constant improvement in the quality of images provided by angiotomography and magnetic resonance angiography, in conjunction with their low invasivity, these newer technologies may gradually substitute traditional arteriography as the gold standard. 11

 Currently, indication of invasive treatment is based on evaluation of a series of factors, such as the type of arterial injury, the extent of renal damage at the time of diagnosis and response to drug-based treatment. 2 In clinical practice, a large proportion of patients are not eligible for invasive treatment because they respond adequately to drug-based treatment. 5 ^,^ 6 ^,^ 9

 Open surgery was used in the majority of series described in the literature. Reilly et al. (1991) achieved a 71.4% success rate with open revascularization over a long follow-up period, with effective control of arterial blood pressure and preservation of renal tissue. 12 Other studies evaluated patients treated surgically. 5 ^,^ 6 ^,^ 9 Although surgery limited the severity of secondary arterial hypertension in all cases, these studies reported high rates of nephrectomy (8-27%), acute renal artery thrombosis (6-12%), and late restenosis (15%). 5 ^,^ 6 ^,^ 9 It was these discouraging results after surgical repair of dissection that led to stent placement being considered the treatment for this pathology. It avoids the need to clamp the renal artery, enabling rapid revascularization with reduced duration of ischemia, and is a less aggressive technique. 

 Endovascular treatment for spontaneous dissection of the renal artery was introduced in 2003 13 with the objective of stabilizing the dissection flap, reopening the true lumen and limiting progression of the subintimal hematoma. Since then, some series have reported encouraging results. 2 In 1989, Mali described successful treatment of a dissecting renal artery aneurysm with stent placement. 14 Long-term follow-up of renal artery dissections treated with stent placement has demonstrated very low or even zero rates of restenosis and satisfactory remodeling of the artery walls. This contrasts with the restenosis rates after placement of stents in renal arteries with atherosclerotic lesions, which are estimated at 15-17% at 2 years. 15 It is possible that stent placement in a dissected artery does not lead to restenosis because of the absence of significant local atherosclerosis. Additionally, a stent placed in a renal artery with no preexisting intimal disease can dramatically reduce migration and proliferation of smooth muscle cells. With regard to technical considerations, it is necessary to completely cover the entire length of the dissection, since reocclusion of the renal artery may be caused by incomplete coverage. 15

 In conclusion, SDRA is more frequent in middle-aged, male smokers, in whom diagnosis is generally delayed because of vague and nonspecific symptoms. Therefore, diagnosis of SDRA is reliant on a high degree of suspicion in patients complaining of abdominal pains in the absence of other specific findings. Angiotomography and magnetic resonance angiography are the methods most frequently used for diagnoses and to choose treatment, which can include drug-based and invasive treatments and should be chosen on a case-by-case basis depending on clinical findings. 
