# Supplementary material for: Spontaneous dissection of the renal artery: case report
Source: J Vasc Bras. 2018 Apr-Jun;17(2):156–9. doi: 10.1590/1677-5449.009917 (PMC6205702; doi:10.1590/1677-5449.009917)
Supplement: Translation

# Translation: Dissecção espontânea da artéria renal: relato de caso

**Authors:** Marcio Miyamotto, Carla Mariko Okabe, Paulo Roberto Pancheniak Neumann, Bruna Da Lozzo, Giana Caroline Strack Neves, Cintia Lopes Raymundo

**Affiliations:** 1 Pontifícia Universidade Católica do Paraná – PUC-PR, Hospital Universitário Cajuru – HUC, Serviço de Cirurgia Vascular e Endovascular, Curitiba, PR, Brasil.; 2 Instituto VESSEL de Aperfeiçoamento Endovascular de Curitiba, Curitiba, PR, Brasil.; 3 Hospital Nossa Senhora das Graças – HNSG, Serviço de Cirurgia Vascular e Endovascular Elias Abrão, Curitiba, PR, Brasil.; 4 Pontifícia Universidade Católica do Paraná – PUC-PR, Hospital Universitário Cajuru – HUC, Liga Acadêmica de Medicina Vascular – LAMEV, Curitiba, PR, Brasil.

**Keywords:** artéria renal, dissecção, angioplastia

## Abstract

A dissecção espontânea da artéria renal é uma patologia rara que, na maioria dos casos, ocorre de forma idiopática. Em alguns casos, pode estar associada à presença de alterações arteriais prévias. Os sintomas geralmente são discretos e inespecíficos, dificultando o diagnóstico. Os autores relatam o caso de um paciente do sexo masculino, de 40 anos, com quadro de hipertensão arterial não controlada de início recente. Na investigação da etiologia da hipertensão arterial, o eco-Doppler de artérias renais revelou a presença de estenose (> 80%) no terço médio da artéria renal esquerda, e a angiografia evidenciou uma dissecção com dupla luz e redução do calibre do vaso. O paciente foi submetido a tratamento com implante de stent com evolução satisfatória.

## INTRODUÇÃO

 A dissecção espontânea da artéria renal (DEAR), descrita inicialmente por Bumpus em 1944 1 , é uma patologia rara, definida como uma dissecção não relacionada a traumas ou intervenções arteriais prévias 2 . Grande parte dos casos acomete indivíduos sem alterações vasculares previamente conhecidas, embora também possa haver associação com aterosclerose, fibroplasia intimal, hipertensão maligna, síndrome de Marfan, síndrome de Ehlers-Danlos, além de esforço físico intenso 3 . 

 Os autores relatam o caso de um paciente com diagnóstico de DEAR não relacionada a alterações renais prévias, manejada de forma endovascular através do implante de stent. 

## RELATO DO CASO

 Paciente do sexo masculino, de 40 anos, apresentou quadro de piora súbita da hipertensão arterial, previamente controlada com duas medicações (anlodipino e valsartana). A função renal permaneceu preservada e sem alterações, assim como os níveis de ureia e creatinina. Apresentava história pregressa de hepatite crônica e tabagismo (carga tabágica: 20 anos/maço). Na investigação de provável hipertensão secundária, foi realizado um eco-Doppler de artérias renais, que revelou a presença de estenose (> 80%) no terço médio da artéria renal esquerda. A imagem apresentada pelo eco-Doppler foi considerada satisfatória e condizente com o quadro clínico do paciente. Além da imagem, os achados do eco-Doppler permitiram calcular a estenose através dos critérios anatômicos e de velocidade, sendo suficientes para indicar angiografia e planejamento terapêutico no mesmo procedimento. Foi realizada então uma angiografia, que evidenciou a dissecção da artéria renal, com dupla luz e redução do calibre do vaso, provocando uma estenose nesse segmento ( Figura 1 ). O paciente foi submetido a tratamento com implante de dois stents, sendo um autoexpansível mais longe e um expansível por balão recoberto ao longo da área de dissecção, com boa evolução clínica e controle da hipertensão arterial com apenas uma medicação. A angiotomografia de controle realizada após três meses evidenciou resolução da dissecção e perviedade dos stents ( Figura 2 ). 

**Figura 1:** Arteriografia evidenciando estenose da artéria renal no terço médio distal.

**Figura 2:** Angiotomografia de controle após implante de stent.

## DISCUSSÃO

 A DEAR pode ocorrer de forma espontânea ou secundária a alterações locais da artéria renal, como aterosclerose, fibroplasia intimal, síndrome de Marfan, síndrome de Ehlers-Danlos, além de hipertensão maligna ou mesmo após intensa atividade física 3 ^,^ 4 . No presente caso, a pesquisa de alterações arteriais compatíveis com displasia fibromuscular nas artérias renais e em outros vasos, bem como de outras doenças vasculares associadas, foi realizada e não indicou a presença de alterações. Embora a etiopatogenia dessa doença permaneça incerta, vários autores sugerem que haveria uma ruptura da camada íntima ou a formação de um hematoma intramural por ruptura de *vasa vasorum* 5 . 

 A DEAR ocorre predominantemente em indivíduos do sexo masculino (a relação homem:mulher pode variar entre 4:1 e 10:1), em geral fumantes entre a quarta e a sexta década de vida 5 ^-^ 8 . O caso do paciente descrito se encaixa nesse perfil. 

 A apresentação clínica da DEAR na fase aguda pode ser inespecífica, manifestando-se como um quadro de dor lombar que pode simular uma cólica renal ou mesmo dores de origem muscular, embora a maioria dos pacientes seja assintomática. A dor também pode ser secundária à ocorrência de infarto renal devido à oclusão abrupta de ramos segmentares ou mesmo da artéria renal principal. Em fases mais tardias, o paciente pode apresentar isquemia renal manifestada por hipertensão renovascular, como no caso descrito 9 . Na anamnese realizada posteriormente para investigar algum fator relacionado com a dissecção da artéria renal, o paciente relatou um episódio de dor lombar inespecífica do lado direito. Na ocasião, relacionou essa dor a uma distensão muscular em decorrência de trauma durante treinamento de luta marcial (jiu-jitsu). Além disso, relatou que o surgimento da dor não estava associado temporalmente aos treinos. Outro dado relevante é que não houve trauma de intensidade suficiente para ocasionar uma dissecção da artéria renal relacionada a traumas contusos. Essa apresentação heterogênea e inespecífica pode confundir e causar atraso no reconhecimento e tratamento dessa entidade, sendo que mais da metade dos pacientes já apresenta infarto renal no momento do diagnóstico 9 . 

 A angiografia é reconhecida como modalidade diagnóstica definitiva nas dissecções da artéria renal, pois demonstra com precisão a extensão e a natureza do envolvimento arterial, além de identificar possíveis alterações associadas 10 . Atualmente, considerando a evolução constante da qualidade das imagens obtidas por angiotomografia e angiorressonância, além de sua baixa invasividade, essas novas tecnologias podem gradualmente substituir a arteriografia tradicional como padrão-ouro 11 . 

 Atualmente, a indicação de tratamento invasivo é baseada em uma avaliação de vários fatores como tipo de lesão arterial, extensão da lesão renal na ocasião do diagnóstico e resposta ao tratamento medicamentoso 2 . Na prática clínica, uma grande parcela de doentes não é elegível para o tratamento invasivo, já que responde de forma adequada ao tratamento medicamentoso 5 ^,^ 6 ^,^ 9 . 

 A cirurgia aberta tem sido o tratamento utilizado na maioria das séries relatadas na literatura. Reilly et al. (1991) obtiveram 71,4% de sucesso na revascularização aberta em um período longo de seguimento, com controle eficiente da pressão arterial e preservação de tecido renal 12 . Outros estudos avaliaram pacientes tratados de forma cirúrgica 5 ^,^ 6 ^,^ 9 . Embora as cirurgias tenham limitado a gravidade da hipertensão arterial secundária em todos os casos, esses estudos mostraram altas taxas de nefrectomia (8-27%), trombose aguda da artéria renal (6-12%) e reestenose tardia (15%) 5 ^,^ 6 ^,^ 9 . Devido aos resultados limítrofes da correção cirúrgica da dissecção, o implante de stent passou a ser considerado o tratamento dessa patologia. Ele evita a necessidade de clampeamento da artéria renal, permitindo uma rápida revascularização que diminui a duração da isquemia, além de ser uma abordagem menos agressiva. 

 O tratamento endovascular da dissecção espontânea da artéria renal foi introduzido em 2003 13 com o objetivo de estabilizar o *flap* de dissecção, reabrir o lúmen verdadeiro e limitar a evolução do hematoma subintimal. Desde então, algumas séries têm mostrado resultados animadores 2 . Em 1989, Mali descreveu o tratamento com implante de stent de um aneurisma dissecante de artéria renal, com sucesso 14 . O seguimento em longo prazo das dissecções da artéria renal tratadas com implante de stent demonstrou índices muito baixos ou mesmo ausência de reestenose com remodelamento satisfatório das paredes arteriais. Isso contrasta com a taxa de reestenose relatada após a colocação do stent da artéria renal em lesões ateroscleróticas, estimada entre 15-17% aos 2 anos 15 . É possível que o implante de stent em uma artéria dissecada não promova a reestenose devido à ausência de aterosclerose significativa local. Além disso, a presença de um stent na artéria renal com ausência de doença pré-existente da íntima pode reduzir de forma dramática a migração e proliferação de células musculares lisas. Considerando a técnica, existe ainda a necessidade de cobrir completamente toda a extensão da dissecção, já que a ocorrência de reoclusão da artéria renal pode ser causada por coberturas incompletas 15 . 

 Em conclusão, a DEAR ocorre mais frequentemente em pacientes fumantes de meia-idade do sexo masculino nos quais o diagnóstico é geralmente atrasado por causa de sintomas vagos e inespecíficos. Portanto, o diagnóstico da DEAR requer um alto índice de suspeita em pacientes que se queixam de dor abdominal sem outros achados específicos. Angiotomografia e angiorressonância são os métodos mais comumente utilizados para o diagnóstico e a escolha de tratamento, incluindo tratamento medicamentoso e invasivo, que deve ser individualizada com base nos achados clínicos.
